# Contrasting patterns of polymorphism and selection in bacterial-sensing toll-like receptor 4 in two house mouse subspecies

**DOI:** 10.1002/ece3.1137

**Published:** 2014-06-20

**Authors:** Alena Fornuskova, Josef Bryja, Michal Vinkler, Miloš Macholán, Jaroslav Piálek

**Affiliations:** 1 Institute of Vertebrate Biology, Academy of Sciences of the Czech RepublicBrno, Czech Republic; 2 Department of Botany and Zoology, Faculty of Science, Masaryk UniversityBrno, Czech Republic; 3 Centre de Biologie pour la Gestion des Populations (CBGP), Institut National de la Recherche Agronomique (INRA), Campus International de BaillarguetMontferrier-sur-Lez, France; 4 Department of Zoology, Faculty of Science, Charles University in PraguePrague, Czech Republic; 5 Laboratory of Mammalian Evolutionary Genetics, Institute of Animal Physiology and Genetics, Academy of Sciences of the Czech RepublicBrno, Czech Republic

**Keywords:** Adaptive evolution, arms race, directional selection, host–pathogen interaction, MAMPs, *Mus musculus*, parasite-mediated selection, pattern-recognition receptors

## Abstract

Detailed investigation of variation in genes involved in pathogen recognition is crucial for understanding co-evolutionary processes between parasites and their hosts. Triggering immediate innate response to invading microbes, Toll-like receptors (TLRs) belong presently among the best-studied receptors of vertebrate immunity. TLRs exhibit remarkable interspecific variation and also intraspecific polymorphism is well documented. In humans and laboratory mice, several studies have recently shown that single amino acid substitution may significantly alter receptor function. Unfortunately, data concerning polymorphism in free-living species are still surprisingly scarce. In this study, we analyzed the polymorphism of Toll-like receptor 4 (*Tlr4*) over the Palearctic range of house mouse (*Mus musculus*). Our results reveal contrasting evolutionary patterns between the two recently (0.5 million years ago) diverged house mouse subspecies: *M. m. domesticus* (Mmd) and *M. m. musculus* (Mmm). Comparison with cytochrome *b* indicates strong directional selection in Mmd *Tlr4*. Throughout the whole Mmd western Palaearctic region, a single variant of the ligand-binding region is spread, encoded mainly by one dominant haplotype (71% of Mmd). In contrast, *Tlr4* in Mmm is much more polymorphic with several haplotypes at intermediate frequencies. Moreover, we also found clear signals of recombination between two principal haplogroups in Mmm, and we identified eight sites under positive selection in our dataset. Our results suggest that observed differences in *Tlr4* diversity may be attributed to contrasting parasite-mediated selection acting in the two subspecies.

## Introduction

Selective forces imposed by parasites can affect various traits of their hosts, including population dynamics, life histories, mating systems, sexual dimorphism etc. (Schmid-Hempel 2011). The detrimental effects of parasites are countered by function of immune system, which in vertebrates comprises both innate and acquired immunity (Danilova 2006). Study of evolution in immune-related genes is, therefore, of paramount importance for comprehension of dynamics in parasite–host relationships (see e.g., Woolhouse et al. 2002; Carlton 2003). Despite the complexity of the immune system, most studies in free-living vertebrates have focused on genes involved in acquired immunity, namely the major histocompatibility complex (MHC; e.g., Milinski 2006). However, mapping and association studies have revealed that at least half of the genetic variation responsible for resistance to various infections is attributable to non-MHC genes (Acevedo-Whitehouse and Cunningham 2006). Most of these genes seem to be associated with innate immunity and there is an increasing evidence that variation in these genes may have a fundamental effect on the host fitness in free-living populations (e.g., Turner et al. 2011; Tschirren et al. 2013).

Innate immunity receptors that directly detect and bind to parasite structures (microbe-associated molecular patterns, MAMP), the pattern-recognition receptors (PRR), stand in the first line of immune defense (Medzhitov and Janeway 2002; Akira et al. 2006). Their fast and effective functioning is thus crucial for host survival (O'Neill 2004; Akira et al. 2006). Among PRRs, the Toll-like receptors (TLR) have been shown to be particularly important (Akira et al. 2001). These receptors form a group of membrane-bound, noncatalytic proteins present in most immune cells, especially in macrophages. Distinct MAMPs (e.g., lipopolysaccharides [LPS] and lipoproteins in bacterial cell walls, zymosan of yeast, bacterial flagellin or viral nucleic acids) are recognized by distinct TLRs and the set of TLR types varies substantially among vertebrate lineages (Janssens and Beyaert 2003; Akira et al. 2006; Vinkler and Albrecht 2009; Kawai and Akira 2010). The potential action of TLRs in the context of host–parasite interactions in free-living organisms is increasingly drawing attention of evolutionary biologists and immunologists (Medzhitov et al. 1997; Pasare and Medzhitov 2004; Takeda and Akira 2005; Vinkler and Albrecht 2009). Contradicting the previous assumption of evolutionary conservatism of these receptors, evolution-focused immunogenetic investigations yielded a clear evidence that at the interspecific level diversifying selection has significantly increased diversity of orthologous *Tlr* genes, mainly in the ligand-binding region (LBR, Poltorak et al. 1998; Smirnova et al. 2000; Downing et al. 2010; Park et al. 2010; Wlasiuk and Nachman 2010; Areal et al. 2011; Tschirren et al. 2011; Fornuskova et al. 2013).

Information regarding the structure and variation of TLRs in free-living rodents is still relatively scarce. Interspecific comparisons of European and Asian rodents confirmed purifying selection as a prevalent evolutionary force shaping these genes (namely *Tlr2, 4,* and *7*), probably due to functional constraints posing on the receptor molecules (Tschirren et al. 2011; Fornuskova et al. 2013). However, signatures of positive selection have also been revealed in all studied genes (mainly in the extracellular domain [ECD], containing LBRs responsible for pathogen recognition, see below), with a more intense signal in the bacterial-sensing *Tlr2* and *Tlr4* than in the viral-sensing *Tlr7* gene (Tschirren et al. 2011; Fornuskova et al. 2013). Following study of Tschirren et al. (2012) showed that TLRs are polymorphic even within species and that intraspecific variation may strikingly differ even between two sympatric species of rodents inhabiting the same environment. In one of these species, the bank vole (*Myodes glareolus*), a particular group of alleles was shown to be significantly associated with resistance to *Borrelia* infection, suggesting an on-going evolution in the receptor (Tschirren et al. 2013). These results illustrate the urgent need for further research focused on polymorphism in PRRs at the intraspecific level, as the genetic variability in PRRs might represent an important missing element for understanding the effects of a host genotype on individual fitness.

TLR4 is one of the most essential bacterial-sensing PRRs, binding, among others, bacterial endotoxins (i.e., LPS) as ligands (Poltorak et al. 1998). At the interspecific level, this cell-surface receptor has the highest number of positively selected sites among all mammalian TLRs (Areal et al. 2011). Most of these sites are localized in the ECD which is responsible for LPS binding (Poltorak et al. 1998; Kim et al. 2007; Vinkler et al. 2009; Fornuskova et al. 2013). This domain consists of several leucine-rich repeat motifs and includes the LBR, which is in direct physical contact with MAMP structures. The ECD is followed by the transmembrane domain (TMD), anchoring the receptor into the cell membrane, and the intracellular domain (ICD). The ICD comprises the Toll/interleukin-1 (TIR) domain responsible for signal transduction and cell activation triggering the immune responses (Werling et al. 2009; Botos et al. 2011).

Genetic research in laboratory mice enabled identification of the *Tlr4* gene function and assessment of the level of its polymorphism among laboratory strains (Poltorak et al. 1998; Smirnova et al. 2000; Stephan et al. 2007). However, artificial genetic variation occurring in “classical” laboratory strains (Yang et al. 2011) hampers understanding variation present in wild mice displaying much wider ranges of immunoresponsivity (Piálek et al. 2008; Abolins et al. 2011; Babayan et al. 2011; Pedersen and Babayan 2011; Riley and Viney 2011). Several house mouse subspecies have been described. Divergence of house mice is usually located to northern India and/or Pakistan and dated to about 0.5 million years ago (Boursot et al. 1993; Suzuki et al. 2004; Geraldes et al. 2008; Macholán et al. 2012). Two subspecies, *M. m. musculus* (Mmm) and *M. m. domesticus* (Mmd), have colonized Europe where they met along a secondary hybrid zone running across the continent (Boursot et al. 1993; Macholán et al. 2007; Bonhomme et al. 2011; Cucchi et al. 2012, 2013). Although the two subspecies might come into contact at least once during the expansions and contractions of their ranges (Duvaux et al. 2011), allowing them to exchange beneficial mutations, they remained for most of the colonization time in allopatry. As their westward expansions followed different routes (Mmm north of the Black Sea, Mmd through the Middle East and Mediterranean region), the two subspecies may have experienced different histories leaving distinct genetic footprints in PRR genes, including *Tlr4*. A recent study of the gastrointestinal tract microbiota in western European mouse populations showed geography to be the most significant factor explaining the composition of bacterial communities in this species (Linnenbrink et al. 2013). Even though gastrointestinal bacteria may have not necessarily been the pathogenic agents selecting for immunological divergence in the two subspecies, we may expect similar geographic or subspecies-specific variation also among other microbes. Genetic differences between non-bacterial parasites of the two house mouse subspecies and the lack of their significant introgression in the hybrid zone have been described recently (Kváč et al. 2013).

In this study, we have analyzed free-living specimens of the two European *Mus musculus* subspecies across a wide geographic range to answer the question whether the distinct recent evolutionary histories of the subspecies have left any footprints in *Tlr4* variation. Based on preliminary data from classical laboratory strains (CLS) and wild-derived strains (WDS), we expect significant differences between the two house mouse subspecies. These potentially contrasting patterns could be explained either by different selection forces mediated by pathogens or simply by differences in demographic histories of the taxa (e.g., population expansions and/or bottlenecks). Given scarcity of data on pathogen background in the sampled regions, we tested the two plausible explanations by analyzing also the mitochondrial cytochrome *b* (*mt-Cytb*) gene widely used as a selectively neutral marker for assessing demographic histories of species and populations. Whereas similar patterns observed in both *mt-Cytb* and *Tlr4* would support the effect of demographic changes, distinct patterns in the two genes would suggest the effect of selection on *Tlr4*. By genotyping *Tlr4* and *mt-Cytb* in 44 Mmd and 30 Mmm sampled across the Western Palaearctic region, we document (1) a subspecies-specific distribution of genetic variation, (2) different selection patterns operating on *Tlr4* gene in the two subspecies, and (3) important role of recombination increasing the polymorphism of the *Tlr4* gene.

## Materials and Methods

### Sampling

**Figure 1:**
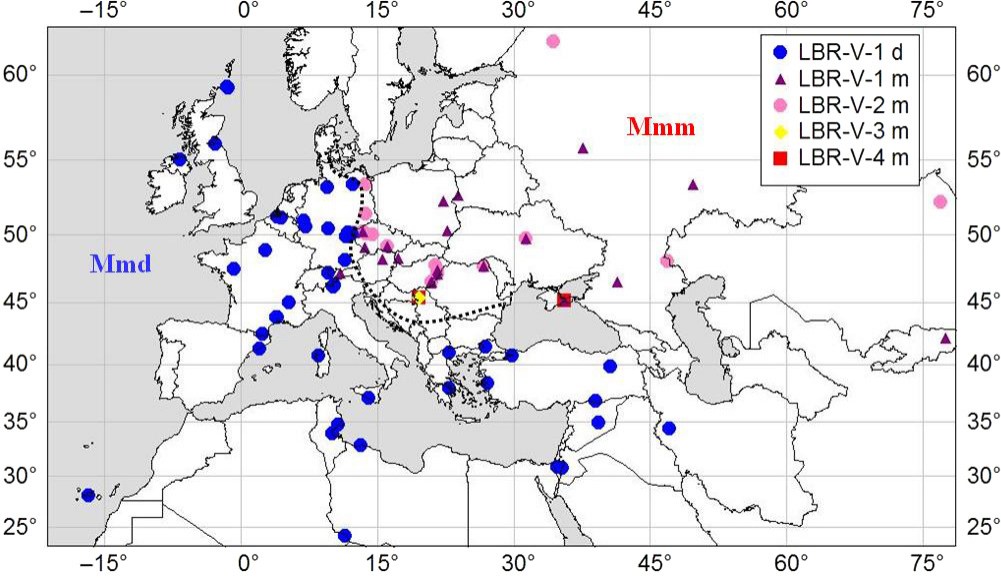
Distribution of samples analyzed in this study. Blue circles: *Mus musculus domesticus* (Mmd); yellow diamond, red squares, violet triangles and pink circles: *M. m. musculus* (Mmm). Different symbols represent distinct protein variants of the ligand-binding region (LBR). Individuals were assigned to the subspecies using hybrid index based on five X-linked loci. Dashed line represents the hybrid zone between two subspecies. Besides sampling localities of free-living populations, the localities of wild-derived strains origin are shown.

We sampled 28 and 42 populations (1–2 individuals per site) of free-living Mmm and Mmd, respectively, scattered across the Western Palaearctic region (with exception of two localities from central Asia; Fig. 1, Table S1). In addition, we included also mice of three CLSs of predominantly Mmd origin (C3Ha, A/J, C57BL/6J; see Yang et al. (2011) for their genomic composition), 15 WDSs of the Mmd origin, and nine WDSs of the Mmm origin (Piálek et al. 2008; Vyskocilová et al. 2009; for the origin of WDSs, see Table S1). In comparison with laboratory strains, WDSs encompass more natural polymorphism and, at the same time, the homozygote variants are useful for distinguishing heterozygote sequences of natural populations (Guénet and Bonhomme 2003; Stephan et al. 2007; Piálek et al. 2008).

### Assignment of specimens to subspecies

Assigning each analyzed individual to one of the two subspecies was based on the combination of X-linked and mtDNA diagnostic markers proven to display low levels of introgression across the European house mouse hybrid zone (Ďureje et al. 2012). The first set of markers consisted of five X-linked SINE and/or LINE insertions chosen to be distributed along the whole chromosome: *X332*, *X65*, *X347*, *Btk*, and *Syap1* (Macholán et al. 2011). For each individual, a hybrid index (HI) was calculated as the mean frequency of Mmm-specific alleles over all five loci (10 for a female and five for a male). While the majority of mice displayed HI = 1.00 (Mmm) or HI = 0.00 (Mmd), 13 individuals were not fixed for all Mmm or all Mmd alleles (Table S1). This may be due to introgression and/or incomplete lineage sorting of the X-linked markers. Note that also C57BL/6, that is, one of the most “classical” laboratory strains of predominately Mmd origin (Yang et al. 2011), harbors Mmm alleles (Table S1). However, regardless underlying causes, in all these cases, admixture was negligible, allowing reliable subspecific identification.

The mitochondrial marker was a *Bam*HI restriction site in the *Nd1* gene shown to discriminate between the subspecies (Božíková et al. 2005). Mice were assigned to Mmm when the site was absent and to Mmd when the site was present. All 62 mice (wild, CLS, and WDS) assigned on the basis of the HI to Mmd also carried the *Bam*HI restriction site. Of the remaining 39 mice assigned to Mmm according to the HI, three individuals (two wild individuals from Lindhorst and Lauchhammer in Germany, and one from a still not fully inbredized WDS established from Lindhorst) carried the Mmd-specific restriction site, suggesting introgression of Mmd mtDNA across the hybrid zone into Mmm range (Table S1). These three specimens (SLINT-WDS, SK843 and SK837) were analyzed as Mmm in the *Tlr4* dataset and as Mmd in the mtDNA dataset (see below for details).

### Genetic variation within subspecies

In total, 101 specimens (free-living mice together with WDSs and CLSs) were successfully sequenced for both *Tlr4* and *mt-Cytb* genes. We sequenced exon 3 of *Tlr4* (2244 bp), encompassing 90% (748 of 835 amino acid residues) of the gene coding sequence, following the protocol described in Fornuskova et al. (2013). Almost complete *mt-Cytb* (1123 bp) was sequenced after amplification by universal primers L14724 and H15915 (Lecompte et al. 2002). Sequences were manually checked and aligned using seqscape v.2.5 (Applied Biosystems, Forster city, CA) and bioedit v.7.1.3 (Hall 1999).

Individual *Tlr4* alleles (thereafter called haplotypes for simplicity of comparison with *mt-Cytb*) were reconstructed from the complete alignment using the Bayesian PHASE routine implemented in DnaSP v.5.10 (Stephens and Donnelly 2003; Librado and Rozas 2009). This analysis was carried out using 1000 iterations, 10 thinning intervals and 1000 burn-in iterations. Four heterozygous *Tlr4* sequences resolved with low support were checked by cloning using the protocol of pGEM®-T Easy Vector System II (Promega Madison, WI). Initially, two clones from each individual were sequenced and this number was later increased until we obtained both sequences of each heterozygote (identification of the four cloned cases can be found in Table S1). Positions of TLR4 domains (ECD, TMD, ICD/TIR) were determined using the on-line program SMART according to Fornuskova et al. (2013). Amino acids were numbered according to a GenBank *M. musculus* TLR4 protein sequence (GenBank Number: AGA16686.1).

The numbers of nucleotide haplotypes (*N*) and amino acid variants (*A*) for both *Tlr4* and *mt-Cytb* genes were estimated using Fabox DNA collapser (Villesen 2007). Nucleotide diversity (*π*), average number of nucleotide differences (*k*), number of polymorphic sites (*S*) and haplotype diversity (Hd) were computed in DnaSP v.5.10. Haplotypes were assigned to haplogroups (HG) based on their phylogenetic interrelationships inferred with MrBayes v. 3.1 (Huelsenbeck and Ronquist 2001) and according to a median joining network constructed with Network v. 4.6.1.1. (Bandelt et al. 1999). The HKY+Γ (Hasegawa et al. 1985) and GTR+Γ (Tavaré 1986) models, determined using jModelTest v. 0.1.1. (Posada 2008), were applied to *Tlr4* and *mt-Cytb* data, respectively. For both genes, we ran 10,000,000 MCMC generations of which 2,500,000 generations were discarded as burn-in. Geographical distribution of the HG was projected onto a map using the PanMap software (http://www.pangaea.de/software/PanMap/). All these computations were based on a subset of wild and WDS mice (i.e., we excluded all sequences from CLSs).

### Analysis of molecular evolution of *Tlr4*

For detection of recombination breakpoints in the *Tlr4* gene, we used two algorithms, the single breakpoint recombination (SBP) and genetic algorithm recombination detection (GARD), provided on the DataMonkey web server (Pond and Frost 2005a,b2005b; Pond et al. 2006a,b2006b; Delport et al. 2010). The *Tlr4* dataset was partitioned according to the breakpoints detected with the SBP and GARD methods. Because it is now widely recognized that the evolutionary process is not homogeneous across sites, we performed also an analysis partitioned by three codon positions.

Selection on *Tlr4* was analyzed at the intersubspecific level. We aimed to identify codons subject to positive or negative selection using test implemented in the DataMonkey program (Pond and Frost 2005a; Pond et al. 2006a): random effects likelihood (REL). The REL test tends to be somewhat susceptible to Type 1 errors, especially for small datasets, where parameter estimates are likely to have large associated errors (Pond and Frost 2005b). The Bayes factor was set up to 50. Finally, we employed the McDonald–Kreitman test (MKT), which compares variation within species to the amount of divergence between species at putatively neutral (synonymous) and nonsynonymous sites (McDonald and Kreitman 1991). Four types of comparisons were used in the MKT: Mmm/Mmd versus rats of the tribe Rattini; Mmm/Mmd versus *R. norvegicus*; Mmm/Mmd versus southeastern-Asian mouse species *M. caroli*, *M. cooki*, *M. cervicolor*; and Mmm versus Mmd (results available upon request). All selection tests were applied to a set of wild and WDS mice (i.e., excluding CLSs). Sequences of Asiatic species of *Mus* and Rattini were taken from Fornuskova et al. (2013).

The crystal structure of mouse TLR4 ECD (PDB 2z64) was adopted and modified from the RCSB PDB Protein Data Bank (http://www.rcsb.org/pdb/explore.do?structureId=2z64; Kim et al. 2007). Subsequently, nonsynonymous substitutions, sites under positive and negative selection detected by REL, and previously described binding sites for LPS and MD-2 (lymphocyte antigen 96; Kim et al. 2007; Park et al. 2009; Ohto et al. 2012) were visualized using PyMOL, v. 1.6 (The PyMOL Molecular Graphics System, Schrödinger, LLC; available at http://www.pymol.org/, accessed January 25, 2013).

## Results

### Genetic diversity of *Tlr4*

We successfully amplified *Tlr4* sequences of 44 wild Mmd (27 homozygotes and 17 heterozygotes) and 30 wild Mmm (17 homozygotes and 13 heterozygotes; see Table S1 for the number of heterozygous sites for each individual). We found neither heterozygotes between Mmd and Mmm subspecific variants nor trans-subspecific polymorphism. Phylogenetic analysis of amplified sequences of both genes (*Tlr4* and *mt-Cytb*) showed divergence of genetic diversity into two clades corresponding to the Mmm and Mmd subspecies (Table S1, Figs. S1, S2). In total, we found 18 and 15 *Tlr4* haplotypes for Mmm and Mmd, respectively (including WDSs, CLSs and wild mice). Similarly, we identified 23 and 37 haplotypes of *mt-Cytb*, for Mmm and Mmd, respectively. All Mmd with the present *Bam*HI restriction site harbored an Mmd-related *mt-Cytb* haplotype, and the same holds for Mmm mice (Table S1, Figs. S1B, S2B).

Genetic variation in the *Tlr4* locus was considerably higher in Mmm (*N*_Mmm_ = 18, *A*_Mmm_ = 15, *π*_Mmm_ = 0.0025 ± 0.00016 SD) than in Mmd (*N*_Mmd_ = 15, *A*_Mmd_ = 7, *π*_Mmd_ = 0. 0009 ± 0.00007 SD). This is even more noticeable for the ECD with fourfold nucleotide diversity and twofold number of segregating sites in Mmm relative to Mmd (Table 1). Contrary to *Tlr4*, genetic variation in *mt-Cytb* was comparable for both subspecies (*N*_Mmm_ = 23, *N*_Mmd_ = 37; *π*_Mmm_ = 0.0047 ± 0.00046 SD, *π*_Mmd_ = 0.0046 ± 0.00022 SD; Table 1).

**Table 1:** Genetic diversity of *Tlr4* and *mt-Cytb* in two house mouse subspecies

	*N/N*^1^	*A/A*^1^	*π* ± SD^1^	*k*^1^	*S*^1^	Hd ± SD^1^
*Tlr*4-exon 3 2244 bp						
Mmd	15/14	7/6	0.0009 ± 0.00007	1.929	10	0.736 ± 0.052
Mmm	18/16	15/13	0.0025 ± 0.00016	5.595	18	0.882 ± 0.028
*Tlr*4-ECD 1644 bp						
Mmd	9/8	5/4	0.0005 ± 0.00007	0.845	6	0.554 ± 0.066
Mmm	12	7/7	0.0020 ± 0.00015	3.267	12	0.800 ± 0.043
*Tlr*4-LBR 666 bp						
Mmd	2/1	2/1	0.0000	0.000	0	0.000
Mmm	7/7	4/4	0.0022 ± 0.00022	1.473	6	0.627 ± 0.063
*Tlr*4-ICD 531 bp						
Mmd	5/5	2/2	0.0020 ± 0.00014	1.085	4	0.568 ± 0.039
Mmm	8/7	8/7	0.0026 ± 0.00016	1.398	4	0.784 ± 0.028
*Tlr*4-TIR 435 bp						
Mmd	4/4	2/2	0.0024 ± 0.00015	1.052	3	0.551 ± 0.038
Mmm	3/2	3/2	0.0001 ± 0.00010	0.047	1	0.047 ± 0.044
*Cyt* b 1123 bp						
Mmd	37/36	15/15	0.0046 ± 0.00022	5.105	49	0.983 ± 0.009
Mmm	23/20	9/9	0.0047 ± 0.00046	5.254	36	0.974 ± 0.016

Mmd, *Mus musculus domesticus*; Mmm, *Mus musculus musculus*; 62 and 39 specimens were analyzed for Mmd and Mmm, respectively; *N*, number of nucleotide haplotypes; *A*, number of amino acid variants; *π*, nucleotide diversity; *k*, average number of nucleotide differences; *S*, number of polymorphic sites; Hd, haplotype diversity; SD, standard deviation.

1 Indicate analysis without wild-derived strains and classical laboratory strains.

**Figure 2:**
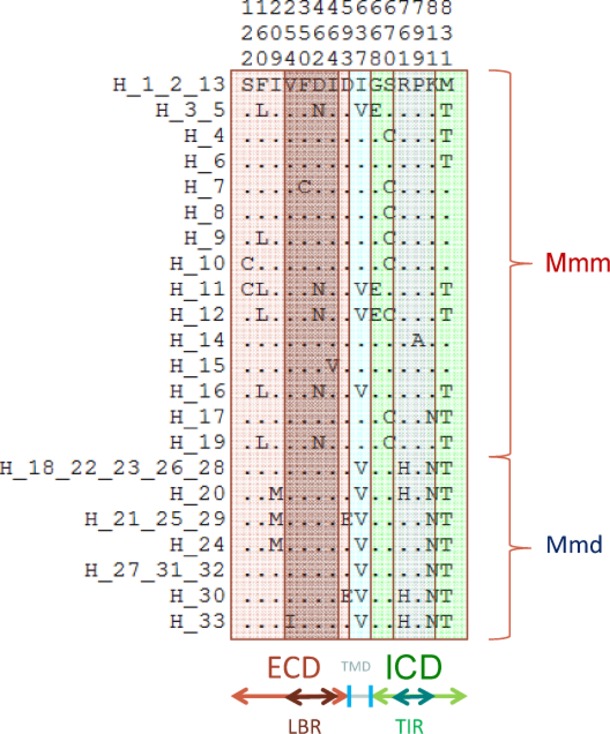
Overview of *Tlr4* nonsynonymous substitutions in Mmd and Mmm. Numbers above alignment indicate amino acid position. ECD, extracellular domain; TMD, transmembrane domain; ICD, intracellular domain; LBR, ligand-binding region; TIR, Toll/interleukin-1 domain. Distribution of individual haplotypes (=alleles) among sampled specimens is presented in Table S1.

Moreover, in all but one Mmd samples, we identified a single protein variant of the LBR. The only exception was the A/J laboratory strain which possessed the conservative substitution V254I. This lack of polymorphism is in contrast to variation in Mmm where four different variants of LBR were found, with two of them being equally frequent in the Mmm distribution area (Fig. 1). These variants differed at three codons (F350C, D462N, and I464V; Table 2). Nevertheless, all substitutions in the LBR brought about exchanges between biochemically similar amino acids. An overview of all amino acid substitutions, their physicochemical properties and distribution are presented in Fig. 2 and Table 3.

**Table 2:** Description of ligand-binding region (LBR) variants. Colored symbols correspond to Fig. 1

LBR variants	I254V	F350C	D462N	I464V
LBR-V-1d 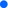	**V**	F	D	I
LBR-V-2d A/J	**I**	F	D	I
LBR-V-1 m 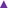	V	**F**	**D**	**I**
LBR-V-2 m 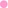	V	**F**	**N**	**I**
LBR-V-3 m 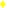	V	**C**	**D**	**I**
LBR-V-4 m 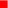	V	**F**	**D**	**V**

The distribution of particular variants among sampled specimens is shown in Table S1.

**Table 3:** Physicochemical properties of the amino acids involved in nonsynonymous substitutions of *Tlr4*

Position	aa1	Properties	aa2	Properties
122	S	SM, P, NEU	C	SM, NP, NEU
160	F	NP, NEU	L	NP, NEU
209	I	NP, NEU	M	NP, NEU
254^1^	V	NP, NEU	I	NP, NEU
350^1^	F	NP, NEU	C	SM, NP, NEU
462^1^	D	SM, P, NEG	N	SM, P, NEU
464^1^	I	NP, NEU	V	NP, NEU
593	D	SM, P, NEG	E	P, NEG
637	I	NP, NEU	V	NP, NEU
668	G	SM, NP, NEU	E	P, NEG
670	S	SM, P, NEU	C	SM, NP, NEU
761^2^	R	P, POS	H	P, POS
799^2^	P	SM, NP, NEU	A	SM, NP, NEU
811^2^	K	P, POS	N	SM, P, NEU
831	M	NP, NEU	T	SM, P, NEU

SM, small; NP, nonpolar; P, polar; NEU, neutral; POS, positively charged; NEG, negatively charged.

1 Sites placed in ligand-binding region.

2 Sites placed in Toll/interleukin-1 domain.

### Haplotype network analysis and distribution of genetic groups

**Figure 3:**
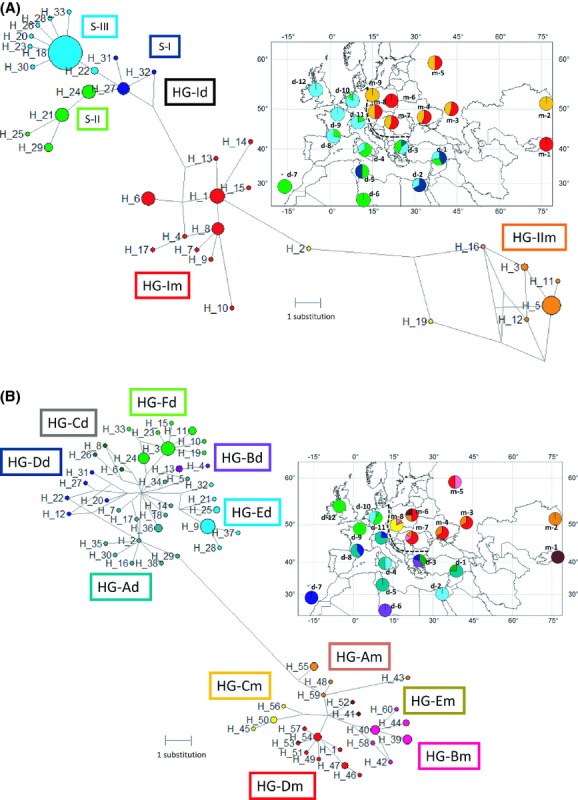
(A) Haplotype network and haplogroup distribution of *Tlr4*, H_haplotypes, HG-, haplogroups, S- subgroups identified in Figs. S1a and S2a. The size of circles corresponds to the frequency of haplotypes; length of lines is related to the number of substitutions. More detailed information can be found in Table S1. The inset figure represents the geographical distribution of HGs. Color circles on the map represent the proportion of particular HG or S (colors correspond to the haplotype network), labels indicate geographic assignment to population groups detailed in Table S1; dashed line shows the position of the house mouse hybrid zone. H_2 and H_19 were excluded from HG due to recombination (see the text for more details). (B) Haplotype network and haplogroup distribution of *mt-Cytb*, H_ identified haplotypes, HG-, identified haplogroup. The size of circles corresponds to the frequency of haplotypes; length of lines is related to the number of substitutions. More detailed information can be found in Table S1. The inset figure represents the geographical distribution of HGs. Color circles on the map represent the proportion of particular HG (colors correspond to the haplotype network); labels indicate geographic assignment to population groups detailed in Table S1; dashed line shows the position of the house mouse hybrid zone.

The haplotype networks based on nucleotide sequences of exon 3 of *Tlr4* were strikingly different in the two mouse subspecies. In Mmd, there was a single most frequent haplotype (H_18; Fig. 3A). It was present in 71% of all individuals (including CLSs and WDSs) and in 66% of wild mice only (in wild mice it was present in 18 specimens in the homozygote state and in 11 specimens as heterozygotes). Conversely, in Mmm, individual haplotypes were more evenly represented, none of them occurring in more than 39% of all specimens. The most common Mmm haplotype (H_5) was found in 33% of wild mice only. Based on the phylogenetic analysis and topology of the haplotype network (Figs. S1, S2), we defined one and two HG for each subspecies, respectively (HG-Idfor Mmd and HG-Im and HG-IIm for Mmm). Notwithstanding the absence of distinct genetic structuring of HG-Id, a subgroup of three haplotypes (for clarity hereafter denoted as S-I) appears rather basal to other two subgroups (S-II and S-III, respectively; Fig. 3A) and restricted to the eastern Mediterranean region and northern Tunisia, while haplotypes of the latter two subgroups either have a wide distribution (e.g., H_18, H_24) or have arisen *in situ* after westward spread of ancestral haplotypes (see the inset in Fig. 3A). This geographic distribution suggests a recent expansion accompanied by a loss of variation. This is especially exemplified by the star-like pattern of S-III haplotypes centered on haplotype H_18 (Fig. 3A).

In Mmm, there were two distinct haplotype clouds separated at least by eight substitutions (HG-Im and HG-IIm). Both groups were interconnected by H_2 (CZ, Buškovice) and H_19 (WDS, DE, Lindhorst) which were not included in any HG (see below). The geographical distribution of HG-Im and HG-IIm is very wide, from central Asia to central Europe and they are largely overlapping in most of the Mmm distribution area. Interestingly, the distance between HG-Im and HG-IIm is higher (minimum eight substitutions) than the distance between HG-Im and HG-Id (minimum four substitutions). In contrast to *Tlr4*, the pattern of the *mt-Cytb* haplotype network was very similar for both subspecies with several star-like branching patterns suggesting local spatial/demographic expansions (Fig. 3B). The geographic distribution of both Mmm and Mmd HG seems to be more intermingled than that of *Tlr4* HGs (see the inset in Fig. 3B). Identification of haplotypes in particular specimens is detailed in Table S1.

### Recombination and selection in the *Tlr4* gene

A recombination breakpoint between Mmd and Mmm at position 1779 bp was detected by both tests implemented in DataMonkey. This breakpoint was recognized in one Mmm individual (ST8335, H_13) sampled in Poland. However, it is based only on a single synonymous substitution at position 849 and homoplasy seems equally plausible explanation. At the intrasubspecific level, we detected recombination in two individuals of Mmm. This breakpoint was identified in a conserved region between the LBR and ICD (the SBP algorithm located the recombination breakpoint to nucleotide position 1587 = AA 529, while GARD placed it to position 1611 = AA 537). Haplotypes H_2 and H_19 likely represent recombinant haplotypes between two main Mmm HG (Fig. S3).

**Figure 4:**
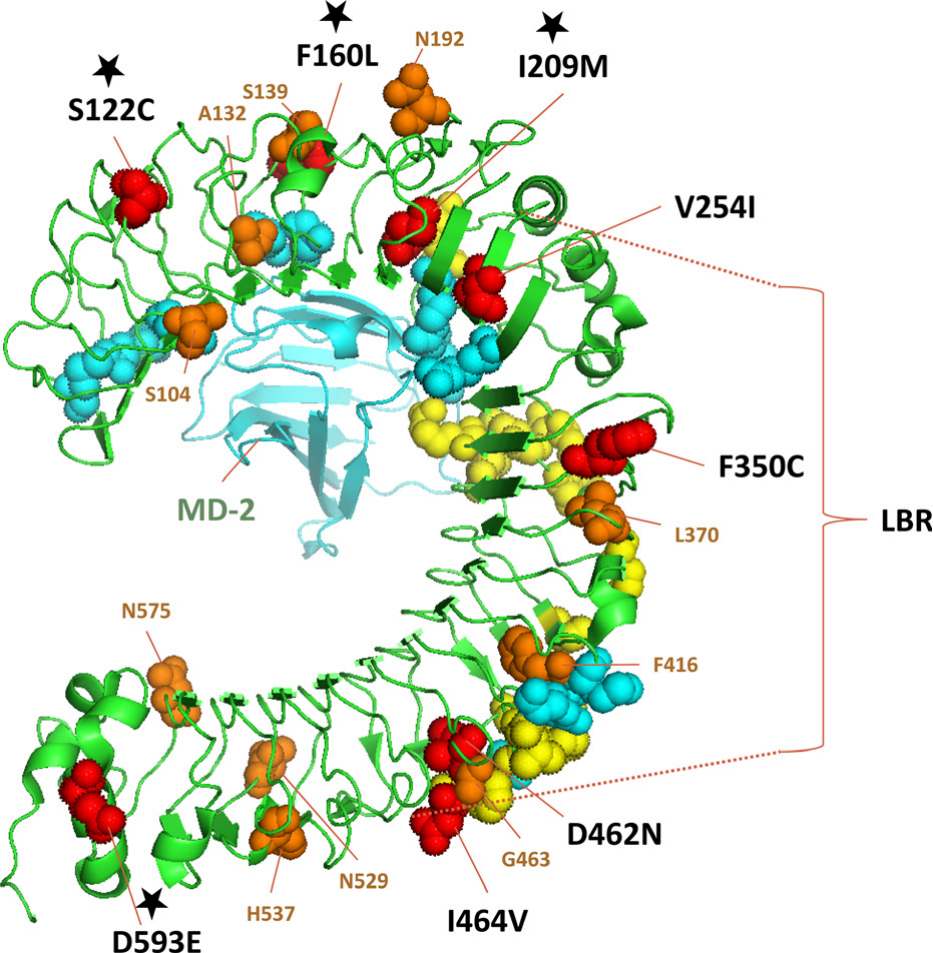
Ribbon diagram of the TLR4 extracellular domain (ECD) 3D structure (PDB 2z64 from RCSB PDB Protein Data Bank, http://www.rcsb.org/pdb/explore.do?structureId=2z64, functional sites were described according to (Kim et al. 2007; Park et al. 2009; Ohto et al. 2012) and important substitutions were visualized as amino acid space-fill models: cyan corresponds to binding positions for MD-2, yellow represents binding sites for lipopolysaccharides (LPS), red represents nonsynonymous amino acid changes, orange represents sites under negative selection detected between subspecies by random effects likelihood (REL); black stars represent detected sites under positive selection differing between the subspecies as revealed by REL; the TLR4 ECD is represented by green color, MD-2 is represented by cyan color, LBR, Ligand-Binding Region is marked by dashed lines. Description of sites responsible for LPS binding and MD-2 binding is in Table S2, modeling of different sites and design correction made in PyMOL Version 1.5.

The REL test detected eight positively and 14 negatively selected sites (Table 4). Four of the positively selected sites were placed in the ECD; however, none of them was in the LBR (Fig. 4, Table 4). Ten of the 14 negatively selected sites were located in the ECD, three of these codons being in LBR (Fig. 4, Table 4). The MK test revealed mostly signs of negative selection (not shown).

**Table 4:** Selection tested by random effects likelihood (REL) in both subspecies together, including wild-derived strains (WDSs); classic laboratory strains (CLSs) were excluded for this analysis

REL (Mmm+Mmd+WDSs)	ECD (88–635)	TMD (636–658)	ICD (659–835)
Positively selected sites	122, 160, 209, 593	637	670, 811, 831
Negatively selected sites	104, 132, 139, 192, 370, 416, 463, 529, 537, 575	647	690, 719, 833

ECD, extracellular domain, TMD, transmembrane domain, ICD, intracellular domain. Underlined sites in ECD are placed in ligand-binding region (248–469). Underlined sites in ICD are placed in Toll/interleukin-1 domain (671–816). Numbers in brackets indicate position of domains in protein (ECD start with codon 88, first 87 codons are in exon 1 and 2). All sites detected by REL had pp = 0.99.

## Discussion

*Tlr*s are generally believed to evolve mainly under purifying selection and, thus, it has been predicted that these genes are relatively uniform within species (e.g., Mukherjee et al. 2009). Contrary to this expectation, we found a moderate intrasubspecific level of *Tlr4* polymorphism. With 15 protein variants in Mmm and 7 protein variants in Mmd, this finding holds true more for Mmm than for Mmd. Indeed, we revealed decreased variation in Mmd *Tlr4* both at the nucleotide and amino acid levels (Table 1), especially in the LBR where we found only a single variant in Mmd, whereas higher polymorphism level (four variants of LBR) is maintained in Mmm populations. Given the crucial function of TLR4 in mammalian innate immune defense, we may assume that the single LBR variant of TLR4 was advantageous in the past, before or during expansion of Mmd into Western Mediterranean and western/north Europe. On the other hand, we observed similar levels and geographic patterns of genetic variation of *mt-Cytb* in both subspecies. This indicates that the observed pattern does not result from a generally decreased level of genetic polymorphism in Mmd. Altogether, our results may imply the action of contrasting types of selection acting specifically on *Tlr4* in the two house mouse subspecies. A similar contrast in selection on TLR4 across geographically distinct populations is known also in other species. For instance, in humans, it has been shown that different haplotypes are positively selected in Sub-Saharan Africa and Eurasia (Ferwerda et al. 2007). Identifying selective forces differentiating subspecies and populations thus appears an intriguing question of current evolutionary biology.

The role of TLR4 in LPS signaling is indisputable and molecular mechanisms of LPS binding were very well described in human and/or mouse (Kim et al. 2007; Park et al. 2009; Resman et al. 2009; Ohto et al. 2012). LPSs are present in the outer membrane of Gram-negative bacteria and immunologically act as endotoxins, that is, substances eliciting a strong immune response in animals. Variability of LPS may affect adhesive properties of a microorganism to the cells of its host but also the induced release of inflammatory mediators. Modifications of LPSs (mainly acylation in the lipid A region) play an important role in the infection process, evasion of the host immune response, and serotypification of Gram-negative bacteria (Robinson et al. 2008). Polymorphism of LPSs has been already shown to be associated with differences in virulence of bacterial strains, for example, *Francisella tularensis, Pseudomonas aeruginosa* or *Yersinia pestis* (Day and Marrceau-Day 1982; Ray et al. 1991; Hajjar et al. 2006; Knirel et al. 2006; Montminy et al. 2006), and as such may be responsible also for evolution and maintenance of recognition mechanisms. This applies especially to *Tlr4* variation. As the genetic variation of human and livestock TLR4 is associated with susceptibility to various infectious and inflammatory diseases (e.g., Leveque et al. 2003; Hawn et al. 2005; Achyut et al. 2007; Sentitula Kumar and Yadav 2012; Zaki et al. 2012) and several nonsynonymous single nucleotide substitutions (nsSNP) has been identified as immunologically relevant (Ferwerda et al. 2007), we focused on physical properties of the nsSNPs we detected in the house mouse *Tlr4*. In total, we detected 15 nsSNP positions, which were distributed evenly across the whole sequenced region including the ECD, TMD, and ICD. Of these 15 nsSNPs we found four (V254I, F350C, D462N and I464V) that were located in the LBR close to the ligand-binding site of LPSs (Fig. 4). Out of these, the substitution V254I has been identified only in the LBR of the A/J laboratory strain and not in any WDS and/or free-living mice (see also Smirnova et al. 2000). We, therefore, suggest that this substitution does not represent a naturally occurring polymorphism and may have originated in laboratory breeds. On the other hand, particularly functionally important might be the residues 462 and 464 that lie in immediate topological proximity to site F461, which has been previously identified as a residuum essential for LPS binding through hydrophobic interactions in mammals (Park et al. 2009; Resman et al. 2009). We, therefore, hypothesize that these nsSNPs can influence the protein function. Our tests of selection, however, did not support this view as no positively selected sites were identified in the LBR. This suggests that D462N and I464V substitutions either have no functional impact or, at least, that there is no selection differentiating these sites in Mmm and Mmd. Nonetheless, the selection analysis showed that three of eight sites positively selected on the intersubspecific level were present in the MD-2-binding region, indicating selection differentiating Mmm and Mmd in the TLR4-MD-2 co-evolution. Recent data have shown that mouse subspecies harbor genetically different parasites (e.g., *Cryptosporidium tyzzeri*; Kváč et al. 2013). Both subspecies may therefore differ in immune response to specific genetic lineages of pathogens. Preliminary laboratory experiments have already shown differences in immunological response between two WDSs derived from both subspecies (Mmm BULS and Mmd STRA) by stimulating *in vitro* by Concanavaline A and a B-cell mitogen bacterial LPS (Piálek et al. 2008).

Although most substitutions identified in the present study involve physically very similar amino acids, it has been shown that even subtle changes in the topological proximity of the binding interface may have substantial impact on the protein function and binding affinity (Zhang et al. 2012). Further studies are, however, needed to test the functional significance of the nsSNPs for recognition of LPS variants.

Previous studies showed that genes encoding TLRs exhibit moderate levels of polymorphism even at intraspecific level (Smirnova et al. 2000; Tschirren et al. 2011; Bergman et al. 2012; Grueber et al. 2012) and that this can have important fitness consequences. In free-living populations, it was documented that selection linked with presence of pathogens can vary across different geographic regions and over time (Tschirren et al. 2012). Polymorphism in immune receptors is thought to be primarily maintained by pathogen-evoked balancing selection. This may be viewed as an evolutionary key-and-lock process described by the Matching alleles model (Frank 1993). Applied to receptor-ligand co-evolution, this model proposes that polymorphism in ligands protecting parasites from recognition is mirrored by adaptive host polymorphism allowing detection of ligand-variants by specifically matching receptor alleles (Agrawal and Lively 2002, 2003).

In addition to nucleotide substitutions, also intragenic recombination can very quickly create new allele variants. In house mouse, the effect of recombination in the evolution of immune genes is well documented, for example, in the MHC genes (Cizkova et al. 2011). However, in most recent studies on intraspecific TLR polymorphism the relevant tests of recombination have not been performed. Using two alternative approaches, our study detected recombination events in the ECD located close to the boundary with the TMD in Mmm. This finding adds another piece of information to the puzzle of PRR polymorphism in free-living rodents showing that recombination might be an important factor increasing TLR variability. Our results are consistent with studies of several other mammals reporting signals of recombination in the ECD in human TLR4 (Zaki et al. 2012) or bovine TLR3, TLR4 and TLR10 (Seabury et al. 2010). Detailed analysis of our sequences suggests that haplotypes H_2 and H_19 are recombinants composed of the ECD from haplogroup HG-IIm and ICD of HG-Im. These two Mmm haplotypes are genetically dissimilar and were found in two specimens separated by 500 km (see Table S1). Assuming that they represent two independent recombination events, we suggest that recombination in this genic region may be relatively frequent in nature. On the other hand, the recombinant haplotypes were found only in two individuals and the estimation of real selective advantage of recombination remains unknown. Because the recombination breakpoints combine different ECDs and ICDs, the WDS SLINT bearing H_19 (in combination with other WDSs from *Tlr4* haplogroups HG-Im and HG-IIm) provides a unique opportunity to discriminate the role of LBR-mediated LPS recognition from the transduction of the signal by the TIR domain.

Although pathogens likely play an important role in evolution of *Tlr4* variability, it may be admitted that the observed difference between the subspecies in TLR4 polymorphism might have arisen as a result of nonadaptive evolutionary processes during mouse colonization of the Western Palearctic. For example, in some avian populations affected by bottlenecks the dominant force influencing evolution of TLRs seems to be genetic drift, outweighing the effect of selection (Grueber et al. 2012, 2013). Similarly, genetic drift also shaped the genetic history of human TLR4 during population expansion out of Africa (Netea et al. 2012). Thus, the pattern observed in mice might result, for example, from differences between subspecies in historical demographic processes (quick expansion of Mmd and two founder populations or refuges for Mmm). In such a case we would, however, expect similar contrasting patterns in *mt-Cytb*. As this was not the case, we may consider the explanation of the observed pattern of *Tlr4* by genetic drift as unlikely. Finally, we must also bear in mind that Mmd *Tlr4* may not be the positively selected gene itself but only a gene involved in gene hitchhiking. Nevertheless, this hypothesis is in contradiction with results of selection analysis, which have detected eight positively selected sites in ECD of free-living *Mus musculus*.

## Supporting Information

**Table S1.** Summary of sampled specimens, identification of haplotypes, and NCBI GenBank accession numbers. **Table S2.** Binding sites between TLR4/LPS/MD-2. **Figure S1.** (A) *Tlr4*, Phylogeny based on Bayesian inference. (B) *mt-Cytb*, Phylogeny based on Bayesian inference. **Figure S2.** (A) *Tlr4*, Haplogroup definition. (B) *mt-Cytb*, Haplogroup definition. **Figure S3.** Evidence of recombination between HG-Im and HG-IIm of Mmm.
