# Supplementary material for: Contrasting patterns of polymorphism and selection in bacterial-sensing toll-like receptor 4 in two house mouse subspecies
Source: Ecol Evol. 2014 Jun 20;4(14):2931–44. doi: 10.1002/ece3.1137 (PMC4130449; doi:10.1002/ece3.1137)

**SUPPORTING INFORMATION**

**Table S1,** Summary of sampled specimens, identification of haplotypes and NCBI GenBank accession numbers.

| **ID** | **Genome HI** | ***Bam*HI = *mt-Cytb*** | **Locality** | **Country** | **Sex** | **G** | **Latitude** | **Longitude** | ***Colour pies, Fig.2 Tlr4*/ *mt-Cytb*** | ***Tlr4*_h1/h2** | ***Tlr4*_LBR-V** | ***mt-Cytb* _h** | ***mt-Cytb* _HG** | **N°of het positions** | **GenBank Acc. *Tlr4*** | **GenBank Acc. *mt-Cytb*** |
| --- | --- | --- | --- | --- | --- | --- | --- | --- | --- | --- | --- | --- | --- | --- | --- | --- |
| **MU103** | 1.00 | - | Tosor | KG | M | 0 | 42° 09' 56" | 77° 26' 40" | m-1 | 14 | 1m | 41 | Em | 0 | KF696929 | KF697033 |
| **SU4736** | 1.00 | - | Petrozavodsk | RU | M | 0 | 61° 47' 00" | 34° 21' 00" | m-5 | 5 | 2m | 54 | Dm | 0 | KF696930 | KF697034 |
| **SU4391** | 1.00 | - | Bakhilova Polyana | RU | M | 0 | 53° 26' 00" | 49° 40' 00" | m-3 | 1 | 1m | 57 | Dm | 0 | KF696931 | KF697035 |
| **SU5214** | 1.00 | - | Sal’sk | RU | M | 0 | 46° 35' 00" | 41° 21' 00" | m-3 | 1 | 1m | 55 | Am | 0 | KF696932 | KF697036 |
| **SU5220** | 1.00 | - | Moscow | RU | M | 0 | 55° 44' 14" | 37° 38' 02" | m-5 | 1 | 1m | 56 | Cm | 0 | KF696933 | KF697037 |
| **SU5218** | 1.00 | - | Shkili Sands, Middle Volga | RU | F | 0 | 48° 08' 00" | 46° 54' 00" | m-3 | 5 | 2m | 54 | Dm | 0 | KF696934 | KF697038 |
| **SK1347** | 1.00 | - | Pavlodar | KZ | F | 0 | 52° 16' 48" | 76° 56' 25" | m-2 | 5 | 2m | 43 | Am | 0 | KF696935 | KF697039 |
| **SU1235** | 1.00 | - | Bialowieza | PL | F | 0 | 52° 42' 00" | 23° 51' 00" | m-2 | 8/1 | 1m | 55 | Am | 1 | KF696936 | KF697040 |
| **ST8337** | 1.00 | - | Majdan Sieniawski | PL | F | 0 | 50° 17' 37" | 22° 43' 10" | m-6 | 8/1 | 1m | 52 | Em | 1 | KF696937 | KF697041 |
| **ST8303** | 1.00 | - | Bielany | PL | F | 0 | 52° 19' 47" | 22° 14' 34" | m-6 | 8/6 | 1m | 46 | Dm | 3 | KF696938 | KF697042 |
| **ST8335** | 1.00 | - | Majdan Sieniawski | PL | F | 0 | 50° 17' 26" | 22° 43' 06" | m-6 | 13 | 1m | 47 | Dm | 0 | KF696939 | KF697043 |
| **SU5158** | 1.00 | - | Bački Petrovac | RS | M | 0 | 45° 20' 29" | 19° 34' 58" | m-7 | 7/8 | 3m/1m | 54 | Dm | 1 | KF696940 | KF697044 |
| **JPC2803** | 1.00 | - | Studenec | CZ | F | 0 | 49° 12' 00" | 16° 04' 00" | m-8 | 5 | 2m | 39 | Bm | 0 | KF696941 | KF697045 |
| **JPC2821** | 1.00 | - | Studenec | CZ | F | 0 | 49° 12' 00" | 16° 04' 00" | m-8 | 6 | 1m | 39 | Bm | 0 | KF696942 | KF697046 |
| **SK1469** | 1.00 | - | Milhostov | CZ | F | 0 | 50° 09' 32'' | 12° 27' 30" | m-8 | 1/5 | 1m/2m | 44 | Bm | 10 | KF696943 | KF697047 |
| **MM1178** | 1.00 | - | Kokrdy | CZ | F | 0 | 50° 06' 41" | 13° 42' 41" | m-8 | 3 | 2m | 40 | Bm | 0 | KF696944 | KF697048 |
| **MM21** | 1.00 | - | Zdíkovec | CZ | F | 0 | 49° 05' 47" | 13° 41' 02" | m-8 | 4 | 1m | 40 | Bm | 0 | KF696945 | KF697049 |
| **SK1004** | 1.00 | - | Buškovice | CZ | M | 0 | 50° 13' 18'' | 13° 22' 27" | m-8 | 1/2 | 1m | 42 | Bm | 3 | KF696946 | KF697050 |
| **SU5607** | 1.00 | - | Stepantsi | UA | M | 0 | 49° 42' 14" | 31° 18' 11" | m-4 | 1/16 | 1m/2m | 47 | Dm | 8 | KF696947 | KF697051 |
| **SU5209** | 1.00 | - | Primorskoe Pheodosiya | UA | M | 0 | 45° 07' 31" | 35° 30' 26" | m-4 | 1/15 | 1m/4m | 55 | Am | 1 | KF696948 | KF697052 |
| **360** | 1.00 | - | Botosani | RO | M | 0 | 47° 45' 00" | 26° 40' 00" | m-4 | 6/5 | 1m/2m | 1 | Dm | 10 | KF696949 | KF697053 |
| **ST8358** | 1.00 | - | Hajdúnánás | HU | F | 0 | 47° 50' 40" | 21° 25' 45" | m-7 | 3/5 | 2m | 53 | Dm | 1 | KF696950 | KF697054 |
| **ST8360** | 1.00 | - | Szepes | HU | F | 0 | 47° 28' 39" | 21° 35' 24" | m-7 | 8/12 | 1m/2m | 48 | Am | 9 | KF696951 | KF697055 |
| **ST8387** | 1.00 | - | Gabortelep | HU | M | 0 | 46° 31' 39" | 20° 54' 59" | m-7 | 8 | 1m | 51 | Dm | 0 | KF696952 | KF697056 |
| **ST8389** | 1.00 | - | Gábortelep | HU | F | 0 | 46° 32' 00" | 20° 56' 00" | m-7 | 9/5 | 1m/2m | 50 | Cm | cloned 10 | KF696953 | KF697057 |
| **ST8381** | 1.00 | - | Szomolyon | HU | F | 0 | 47° 07' 43" | 21° 34' 08" | m-7 | 10/11 | 1m/2m | 49 | Dm | 10 | KF696954 | KF697058 |
| **SK18** | 0.80 | - | Staudach | AT | F | 0 | 47° 16' 17" | 10° 57' 23" | m-8 | 6 | 1m | 45 | Cm | 0 | KF696955 | KF697059 |
| **SK1787** | 1.00 | - | Thallern | AT | F | 0 | 48° 17' 08" | 15° 33' 11" | m-8 | 6 | 1m | 50 | Cm | 0 | KF696956 | KF697060 |
| **SK843** | 1.00 | + | Lindhorst | DE | M | 0 | 53° 26' 34" | 13° 46' 06" | m-9/d-10 | 5 | 2m | 9 | Ed | 0 | KF696957 | KF697031 |
| **SK837** | 0.70 | + | Lauchhammer | DE | F | 0 | 51° 28' 06" | 13° 44' 24" | m-9/d-10 | 5 | 2m | 9 | Ed | 0 | KF696958 | KF697032 |
| **STUF-WDS** | 1.00 | - | Studenec | CZ | F,M | 31 | 49° 12' 00" | 16° 04' 00" | m-8 | 6 | 1m | 60 | Bm | 0 | KF696959 | KF697061 |
| **STUP-WDS** | 1.00 | - | Studenec | CZ | F,M | 30 | 49° 12' 00" | 16° 04' 00" | m-8 | 5 | 2m | 39 | Bm | 0 | KF696960 | KF697062 |
| **STUS-WDS** | 1.00 | - | Studenec | CZ | F,M | 22 | 49° 12' 00" | 16° 04' 00" | m-8 | 6 | 1m | 39 | Bm | 0 | KF696961 | KF697063 |
| **BULS-WDS** | 1.00 | - | Buškovice | CZ | F,M | 25 | 50° 13' 18" | 13° 22' 27" | m-8 | 5 | 2m | 58 | Bm | 0 | KF696962 | KF697064 |
| **BUSNA-WDS** | 1.00 | - | Buškovice | CZ | F,M | 30 | 50° 13' 18" | 13° 22' 27" | m-8 | 5 | 2m | 40 | Bm | 0 | KF696963 | KF697065 |
| **PWD-WDS** | 1.00 | - | Kunratice | CZ | F,M | x+12 | 50° 00' 47" | 14° 29' 07" | m-8 | 5 | 2m | 40 | Bm | 0 | KF696964 | KF697066 |
| **SENK-WDS** | 1.00 | - | Šenkvice | SK | F,M | 1 | 48° 17' 55" | 17° 21' 05" | m-8 | 17 | 1m | 59 | Am | 0 | KF696965 | KF697067 |
| **SMIL-WDS** | 1.00 | - | Milhostov | CZ | F,M | 3 | 50° 09' 33" | 12° 27' 30" | m-8 | 5 | 2m | 44 | Bm | 0 | KF696966 | KF697068 |
| **SLINT-WDS** | 1.00 | + | Lindhorst | DE | F,M | 1 | 53° 26' 34" | 13° 46' 06" | m-9/d-10 | 19 | 2m | 9 | Ed | 0 | KF696967 | KF697030 |
| **SHY-WDS** | 0.00 | + | Hohenberg | DE | F,M | 4 | 50° 06' 07" | 12° 12' 46" | d-10 | 18 | 1d | 9 | Ed | 0 | KF696867 | KF696968 |
| **STRA-WDS** | 0.00 | + | Straas | DE | F,M | 29 | 50° 10' 53" | 11° 45' 44" | d-10 | 18 | 1d | 3 | Fd | 0 | KF696868 | KF696969 |
| **STRB-WDS** | 0.00 | + | Straas | DE | F,M | 26 | 50° 10' 53" | 11° 45' 44" | d-10 | 18 | 1d | 3 | Fd | 0 | KF696869 | KF696970 |
| **STLT-WDS** | 0.00 | + | Straas | DE | F,M | 19 | 50° 10' 53" | 11° 45' 44" | d-10 | 18 | 1d | 3 | Fd | 0 | KF696870 | KF696971 |
| **SCHUNT-WDS** | 0.00 | + | Schweben | DE | F,M | 7 | 50° 26' 00" | 09° 35' 00" | d-10 | 18 | 1d | 9 | Ed | 0 | KF696871 | KF696972 |
| **SCHEST-WDS** | 0.00 | + | Schweben | DE | F,M | 7 | 50° 26' 10" | 09° 35' 10" | d-10 | 18 | 1d | 9 | Ed | 0 | KF696872 | KF696973 |
| **SIN-WDS** | 0.00 | + | Scar, Sanday Isl., Orkneys | UK | F,M | 5 | 59° 18' 00" | -02° 33' 00'' | d-12 | 18 | 1d | 24 | Fd | 0 | KF696873 | KF696974 |
| **SIT-WDS** | 0.00 | + | Scar, Sanday Isl., Orkneys | UK | F,M | 6 | 59° 18' 00" | -02° 33' 00'' | d-12 | 18 | 1d | 24 | Fd | 0 | KF696874 | KF696975 |
| **SFEL-WDS** | 0.00 | + | Feldkirch | AT | F,M | 2 | 47° 15' 42" | 09° 35' 10'' | d-10 | 18 | 1d | 38 | Ad | 0 | KF696875 | KF696976 |
| **SPOS-WDS** | 0.00 | + | Migiondo | IT | F,M | 3 | 46° 19' 23" | 10° 18' 17'' | d-11 | 18 | 1d | 36 | Ad | 0 | KF696876 | KF696977 |
| **SUV-WDS** | 0.00 | + | Sernio | IT | F,M | 3 | 46° 13' 26" | 10° 12' 20'' | d-11 | 18 | 1d | 36 | Ad | 0 | KF696877 | KF696978 |
| **SCHEFE-WDS** | 0.00 | + | Schweben | DE | F,M | 7 | 50° 26' 10" | 09° 35' 10'' | d-10 | 18 | 1d | 9 | Ed | 0 | KF696878 | KF696979 |
| **STAIL-WDS** | 0.00 | + | Schweben | DE | F,M | 9 | 50° 26' 00" | 09° 35' 00'' | d-10 | 18 | 1d | 9 | Ed | 0 | KF696879 | KF696980 |
| **SOTT-WDS** | 0.00 | + | Ottmannsreuth | DE | F,M | 1 | 49° 53' 27" | 11° 37' 04'' | d-10 | 18 | 1d | 11 | Fd | 0 | KF696880 | KF696981 |
| **SPLY-WDS** | 0.00 | + | Plössen | DE | F,M | 2 | 49° 51' 18" | 11° 47' 10" | d-10 | 18 | 1d | 11 | Fd | 0 | KF696881 | KF696982 |
| **C57BL/6J-CLS** | 0.20 | + | classical laboratory strain | lab | F,M | - | - | - | - | 29 | 1d | 3 | Fd | 0 | KF696882 | KF696983 |
| **A/J-CLS** | 0.00 | + | classical laboratory strain | lab | F,M | - | - | - | - | 33 | 2d | 3 | Fd | 0 | KF696883 | KF696984 |
| **C3Ha-CLS** | 0.00 | + | classical laboratory strain | lab | F,M | - | - | - | - | 29 | 1d | 3 | Fd | 0 | KF696884 | KF696985 |
| **SK1482** | 0.00 | + | Plössen | DE | F | 0 | 49° 51' 18" | 11° 47' 10" | d-10 | 18 | 1d | 11 | Fd | 0 | KF696885 | KF696986 |
| **JPC2705** | 0.00 | + | Straas | DE | F | 0 | 50° 10' 53" | 11° 45' 44" | d-10 | 18 | 1d | 3 | Fd | 0 | KF696886 | KF696987 |
| **ST5068** | 0.00 | + | Arzdorf | DE | F | 0 | 50° 36' 00" | 07° 05' 00" | d-10 | 18 | 1d | 21 | Ed | 0 | KF696888 | KF696989 |
| **ST6688** | 0.00 | + | Arzdorf | DE | F | 0 | 50° 36' 00" | 07° 05' 00" | d-10 | 18/23 | 1d | 3 | Fd | 1 | KF696891 | KF696992 |
| **SU607** | 0.00 | + | Schweben | DE | F | 0 | 50° 26' 00" | 09° 35' 00" | d-10 | 18 | 1d | 9 | Ed | 0 | KF696887 | KF696988 |
| **SU627** | 0.00 | + | Schweben | DE | F | 0 | 50° 26' 10" | 09° 35' 10" | d-10 | 18 | 1d | 37 | Ed | 0 | KF696893 | KF696994 |
| **SK6** | 0.10 | + | München | DE | F | 0 | 48° 09' 00" | 11° 26' 00" | d-10 | 24 | 1d | 16 | Ad | 0 | KF696889 | KF696990 |
| **SK899** | 0.00 | + | Hamersen | DE | M | 0 | 53° 15' 15" | 09° 28' 50" | d-10 | 18 | 1d | 19 | Fd | 0 | KF696890 | KF696991 |
| **ST7519** | 0.00 | + | Köln | DE | F | 0 | 50° 58' 37" | 06° 57' 18" | d-10 | 18 | 1d | 23 | Fd | 0 | KF696892 | KF696993 |
| **SK957** | 0.00 | + | Suckow | DE | F | 0 | 53° 24' 44" | 12° 19' 40" | d-10 | 18 | 1d | 9 | Ed | 0 | KF696894 | KF696995 |
| **ST9613** | 0.00 | + | Scar, Whitemill Bay, Sanday Isl. | UK | F | 0 | 59° 18' 00" | -02° 33' 00" | d-12 | 18 | 1d | 24 | Fd | 0 | KF696895 | KF696996 |
| **ST9597** | 0.00 | + | Bay of Brough, Sanday Isl. | UK | F | 0 | 59° 16' 00" | -02° 36' 00" | d-12 | 18 | 1d | 24 | Fd | 0 | KF696896 | KF696997 |
| **ST9600** | 0.00 | + | Little Sea, Sanday Isl. | UK | F | 0 | 59° 15' 00" | -02° 35' 00" | d-12 | 18/20 | 1d | 24 | Fd | 1 | KF696897 | KF696998 |
| **SK2046** | 0.00 | + | Edinburgh | UK | M | 0 | 55° 57' 03" | -03° 10' 53" | d-12 | 18 | 1d | 15 | Fd | 0 | KF696898 | KF696999 |
| **SU5122** | 0.00 | + | Londonderry | IE(UK) | F | 0 | 55° 00' 25" | -07° 17' 20" | d-12 | 18 | 1d | 24 | Fd | 0 | KF696899 | KF697000 |
| **SU4648** | 0.00 | + | Antwerp | BE | M | 0 | 51° 11' 50" | 04° 24' 35" | d-10 | 29/30 | 1d | 28 | Ed | 4 | KF696900 | KF697001 |
| **SK1349** | 0.00 | + | Stekene | BE | M | 0 | 51° 13' 53" | 04° 00' 33" | d-10 | 21/18 | 1d | 10 | Fd | 4 | KF696901 | KF697002 |
| **SK1515** | 0.00 | + | Saint Jean-et-Royans | FR | F | 0 | 45° 01' 10" | 05° 16' 20" | d-8 | 18 | 1d | 12 | Dd | 0 | KF696902 | KF697003 |
| **ST7605** | 0.00 | + | Brouzet-les-Quissac | FR | F | 0 | 43° 50' 00" | 03° 58' 00" | d-8 | 21/22 | 1d | 35 | Ad | 3 | KF696903 | KF697004 |
| **SU5046** | 0.00 | + | Lagny | FR | F | 0 | 48° 52' 00" | 02° 43' 00" | d-9 | 18 | 1d | 33 | Fd | 0 | KF696904 | KF697005 |
| **SU4886** | 0.00 | + | Pi de Conflent | FR | F | 0 | 42° 30' 00" | 02° 21' 00" | d-8 | 18/26 | 1d | 29 | Ad | 1 | KF696905 | KF697006 |
| **JPC2912** | 0.00 | + | Valflaunes | FR | F | 0 | 43° 48' 00" | 03° 52' 00" | d-8 | 21/18 | 1d | 2 | Ad | 4 | KF696906 | KF697007 |
| **SU5313** | 0.00 | + | Les Brosses | FR | F | 0 | 47° 30' 26" | -00° 46' 03" | d-9 | 18 | 1d | 3 | Fd | 0 | KF696907 | KF697008 |
| **SER1047** | 0.00 | + | Sernio | IT | F | 0 | 46° 13' 26" | 10° 12' 20" | d-11 | 18 | 1d | 36 | Ad | 0 | KF696908 | KF697009 |
| **SU5019** | 0.10 | + | Falconara, Sicily | IT | F | 0 | 37° 06' 32" | 14° 02' 16" | d-4 | 24/18 | 1d | 32 | Ed | 3 | KF696909 | KF697010 |
| **SU4894** | 0.00 | + | Sardinia | IT | M | 0 | 40° 43' 00" | 08° 35' 00" | d-4 | 21 | 1d | 30 | Ad | 0 | KF696910 | KF697011 |
| **ST8495** | 0.10 | + | Tovo S Agata | IT | F | 0 | 46° 14' 32" | 10° 14' 33" | d-11 | 21/22 | 1d | 22 | Dd | 3 | KF696911 | KF697012 |
| **SU4991** | 0.00 | + | Alojera, Canary Archipel. | ES | M | 0 | 28° 09' 00" | -17° 19' 00" | d-7 | 25 | 1d | 31 | Dd | 0 | KF696912 | KF697013 |
| **SK916** | 0.00 | + | El Prat del Llobregat | ES | M | 0 | 41° 18' 00" | 02° 04' 00" | d-8 | 18 | 1d | 20 | Dd | 0 | KF696913 | KF697014 |
| **MM760** | 0.00 | + | Kilkis | GR | M | 0 | 40° 59' 42" | 22° 52' 14" | d-3 | 21/18 | 1d | 6 | Cd | cloned 4 | KF696914 | KF697015 |
| **R307** | 0.10 | + | Korinthos | GR | F | 0 | 37° 56' 19" | 22° 55' 43" | d-3 | 27 | 1d | 13 | Bd | 0 | KF696915 | KF697016 |
| **TU21** | 0.00 | + | Harran | TR | F | 0 | 36° 51' 00" | 39° 01' 00" | d-1 | 27/24 | 1d | 34 | Ad | 1 | KF696916 | KF697017 |
| **SK1763** | 0.20 | + | Kuleli | TR | M | 0 | 41° 29' 39" | 26° 56' 31" | d-3 | 18 | 1d | 13 | Bd | 0 | KF696917 | KF697018 |
| **SU3770** | 0.00 | + | Izmir | TR | F | 0 | 38° 24' 07" | 27° 07' 12" | d-3 | 24/18 | 1d | 27 | Dd | 3 | KF696918 | KF697019 |
| **SK1768** | 0.00 | + | Gölcük | TR | M | 0 | 40° 42' 55" | 29° 49' 37" | d-3 | 24/18 | 1d | 8 | Cd | 3 | KF696919 | KF697020 |
| **SU6834** | 0.00 | + | Ciftlik, Askale | TR | M | 0 | 39° 47' 11" | 40° 38' 31" | d-1 | 24/18 | 1d | 26 | Cd | 3 | KF696920 | KF697021 |
| **SU5229** | 0.20 | + | Hazeva | IL | F | 0 | 30° 46' 02" | 35° 16' 39" | d-2 | 31 | 1d | 25 | Ed | 0 | KF696921 | KF697022 |
| **SU5224** | 0.20 | + | Nachal Nizzana | IL | F | 0 | 30° 51' 30" | 34° 45' 05" | d-2 | 28/27 | 1d | 25 | Ed | cloned 3 | KF696922 | KF697023 |
| **SK1773** | 0.00 | + | Kermanshah | IR | M | 0 | 34° 23' 00" | 47° 06' 00" | d-1 | 27 | 1d | 14 | Ad | 0 | KF696923 | KF697024 |
| **M273** | 0.00 | + | Palmyra | SY | M | 0 | 34° 56' 00" | 39° 16' 00" | d-1 | 32/18 | 1d | 5 | Ad | cloned 3 | KF696924 | KF697025 |
| **SK811** | 0.20 | + | Gabes | TN | M | 0 | 33° 53' 35" | 10° 06' 06" | d-5 | 27 | 1d | 17 | Ad | 0 | KF696925 | KF697026 |
| **SK813** | 0.20 | + | Sfax | TN | M | 0 | 34° 45' 52" | 10° 45' 11" | d-5 | 21/27 | 1d | 18 | Ad | 2 | KF696926 | KF697027 |
| **PB1599** | 0.20 | + | Al Qusbat | LY | M | 0 | 32° 53' 00" | 13° 09' 00" | d-5 | 21 | 1d | 7 | Ad | 0 | KF696927 | KF697028 |
| **LIB11** | 0.20 | + | Al Awayna | LY | M | 0 | 24° 10' 00" | 11° 30' 00" | d-6 | 24 | 1d | 4 | Bd | 0 | KF696928 | KF697029 |

**NOTE. ID** - identification of specimens, **WDS** - wild-derived strain; C**LS** - laboratory strain; **Genome** – classification of mice based on hybrid index, **HI**, estimated from 5 X-linked loci (for details see Methods) to either subspecies (ranging from 0.00 for *Mus musculus domesticus* to 1.00 for *M. m. musculus*); ***Bam*HI** - presence (+) or absence (-) of restriction site; **Country codes**: **AT** - Austria, **CZ** - Czech Republic, **BE** - Belgium, **DE** - Germany, **ES** - Spain, **FR** - France, **GR** - Greece, **HU** - Hungary, **IT** - Italy, **IE** - Northern Ireland, **IL** - Israel, **IR** - Iran, **KG** - Kyrgyzstan, **KZ** - Kazakhstan, **LY** - Libyan Arab Jamahiriya, **PL** - Poland, **RO** - Romania, **RS** - Serbia, **RU** - Russian Federation, **SK** - Slovakia, **SY** - Syrian Arab Republic, **TN** - Tunisia, **TR** - Turkey, **UA** - Ukraine, **UK** - United Kingdom; **NCS** - non-classified sample; **G** – number of generation under brother x sister matings, G0 stands for wild mice, x+ indicates that the strain was kept for some generations in different laboratory; **Colour pies, Fig.2 *Tlr4*/*mt-Cytb*,** labels m-1-9 and d-1-12 correspond to pies at **Fig. 2a** and **2b**, discrepancies between *Tlr4* and *mt-Cytb* are represented by **/**; ***Tlr4*_h1/h2** -haplotype identification, doubled labels indicate heterozygotes;  ***Tlr4*_LBR-V** – indicates type of LBR variants, two variants are marked by /, labels correspond to **Fig. 1** and **Table 2**; ***mt-Cytb*_h** - cytochrome b haplotype identification; ***mt-Cytb*_HG** -cytochrome b haplogroups identification; **N°of het positions** - number of heterozygous sites for *Tlr4*; **GenBank Acc.** - GenBank Accession numbers.

**Table S2,** Binding sites between TLR4/LPS/MD-2, based on knowledge of 3D-crystalography in human (predicted by Kim et al. 2007; Park et al. 2009; Resman et al. 2009; Ohto et al. 2012); substitutions with suggested key role in ligand binding interactions are in bold, substitutions with questioned role are in grey. Exon 2 was not analyzed in our study.

| Position in mus | Localization | Numbering in human sequence | Function of AA | Publication |
| --- | --- | --- | --- | --- |
| C28 | exon2 | hTLR4_C29 | key sites for MD-2 dimerization, documented in human | Nishitani et al. 2006 |
| C39 | exon2 | hTLR4_C40 | key sites for MD-2 dimerization, documented in human | Nishitani et al. 2006 |
| D41 | exon2 | hTLR4_E42 | key sites for MD-2 dimerization | Kim et al. 2007 |
| D83 | exon2 | hTLR4_D84 | key sites for MD-2 dimerization | Kim et al. 2007 |
| E134 | exon3 | hTLR4_E135 | key sites for MD-2 dimerization | Kim et al. 2007 |
| H158 | exon3 | hTLR4_H159 | key sites for MD-2 dimerization | Kim et al. 2007 |
| R233 | exon3 | hTLR4_R234 | key sites for MD-2 dimerization | Kim et al. 2007 |
| **K263** | exon3 | hTLR4_R264 | LPS (Charge interaction with phosphates) | Park et al. 2009 |
| R266 | exon3 | hTLR4_G267 | Residues Involved in the Species Specificity for Lipid IVa | Ohto et al. 2012 |
| R288 | exon3 | hTLR4_R300 | key sites for MD-2 dimerization | Kim et al. 2007 |
| K319 | exon3 | hTLR4_E321 | Residues Involved in the Species Specificity for Lipid IVa | Ohto et al. 2012 |
| R337 | exon3 | hTLR4_N339 | Residues Involved in the Species Specificity for Lipid IVa | Ohto et al. 2012 |
| Q339 | exon3 | hTLR4_K341 | LPS (Charge interaction with phosphates) | Park et al. 2009 |
| K341 | exon3 | hTLR4_G343 | Residues Involved in the Species Specificity for Lipid IVa | Ohto et al. 2012 |
| **K360** | exon3 | hTLR4_K362 | LPS (Charge interaction with phosphates) | Park et al. 2009 |
| K367 | exon3 | hTLR4_E369 | Residues Involved in the Species Specificity for Lipid IVa | Ohto et al. 2012 |
| S386 | exon3 | hTLR4_K388 | LPS (Charge interaction with phosphates) | Park et al. 2009, Resman et al. 2009 |
| S413 | exon3 | hTLR4_ S415 | LPS, hydrogen bond with 1-PO4 | Ohto et al. 2012 |
| A414 | exon3 | hTLR4_ S416 | MD-2 (Hydrogen bond) | Park et al. 2009, Ohto et al. 2012 |
| N415 | exon3 | hTLR4_ N417 | MD-2 (Hydrogen bond) | Park et al. 2009, Ohto et al. 2012 |
| M417 | exon3 | hTLR4_ L419 | MD-2 (Hydrophobic interaction) | Ohto et al. 2012 |
| **R434** | exon3 | hTLR4_Q436 | LPS , MD-2 (Hydrogen bond) | Park et al. 2009 |
| **E437** | exon3 | hTLR4_E439 | MD-2 (Hydrogen bond) | Park et al. 2009 |
| **F438** | exon3 | hTLR4_F440 | LPS, MD-2 (Hydrophobic interaction) | Park et al. 2009, Resman et al. 2009 |
| L442 | exon3 | hTLR4_L444 | LPS, MD-2 (Hydrophobic interaction) | Park et al. 2009, Resman et al. 2009 |
| S443 | exon3 | hTLR4_ S445 | MD-2 (Hydrophobic interaction) | Ohto et al. 2012 |
| **F461** | exon3 | hTLR4_F463 | LPS, MD-2 (Hydrophobic interaction) | Park et al. 2009, Resman et al. 2009 |

**Fig. S1-a**, *Tlr4* phylogeny based on Bayesian inference (MrBayes v.3.1), 10,000,000 iterations and 2,500,000 burn-in iterations, distribution of haplotypes among individuals can be found in Table S1. Numbers above branches indicate posterior support of particular branch.


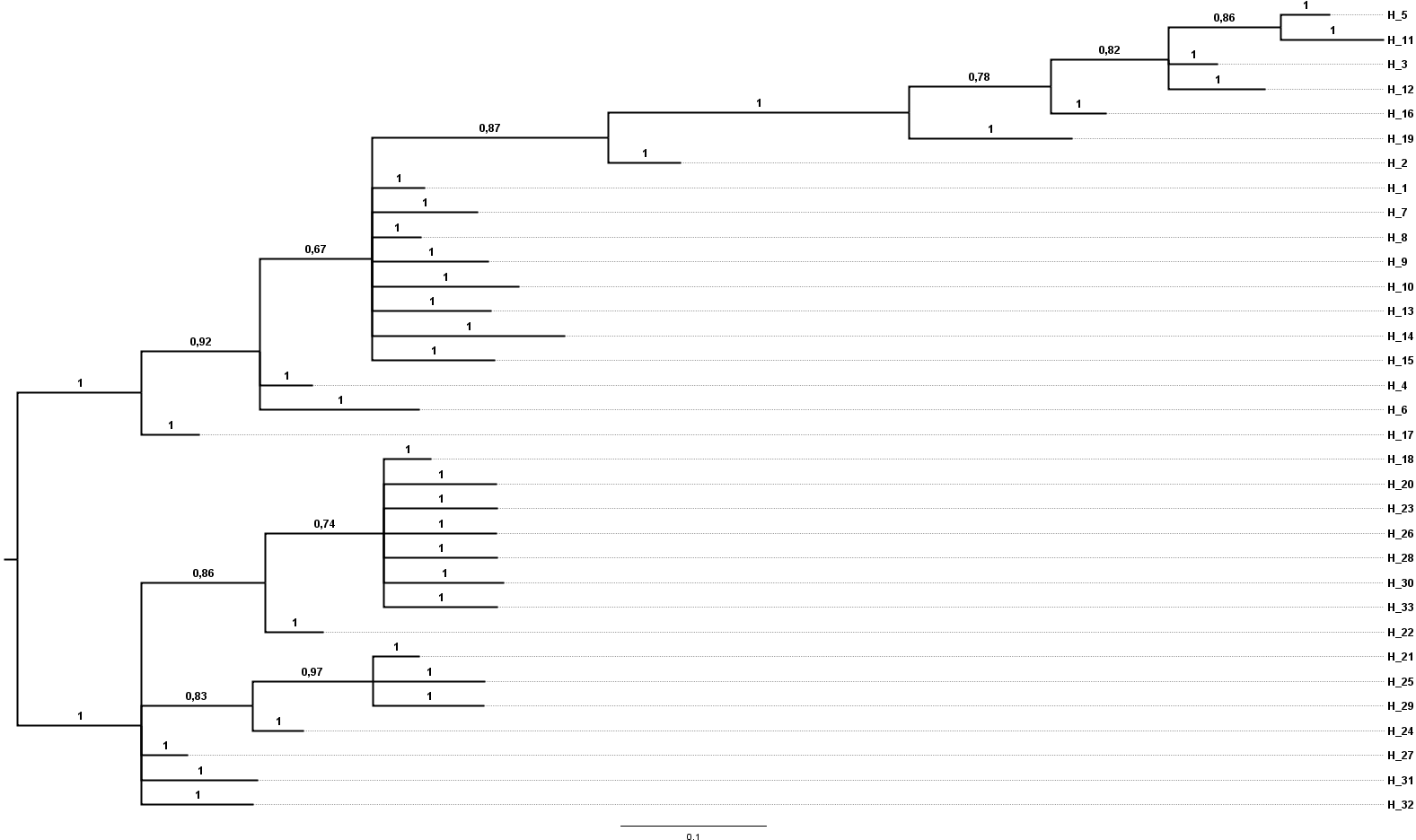


**Fig. S1-b**, *mt-Cytb* phylogeny was based on Bayesian inference (MrBayes v.3.1), 10,000,000 iterations and 2,500,000 burn-in iterations; distribution of haplotypes among individuals can be found in Table S1. Numbers above branches indicate posterior support of particular branch.


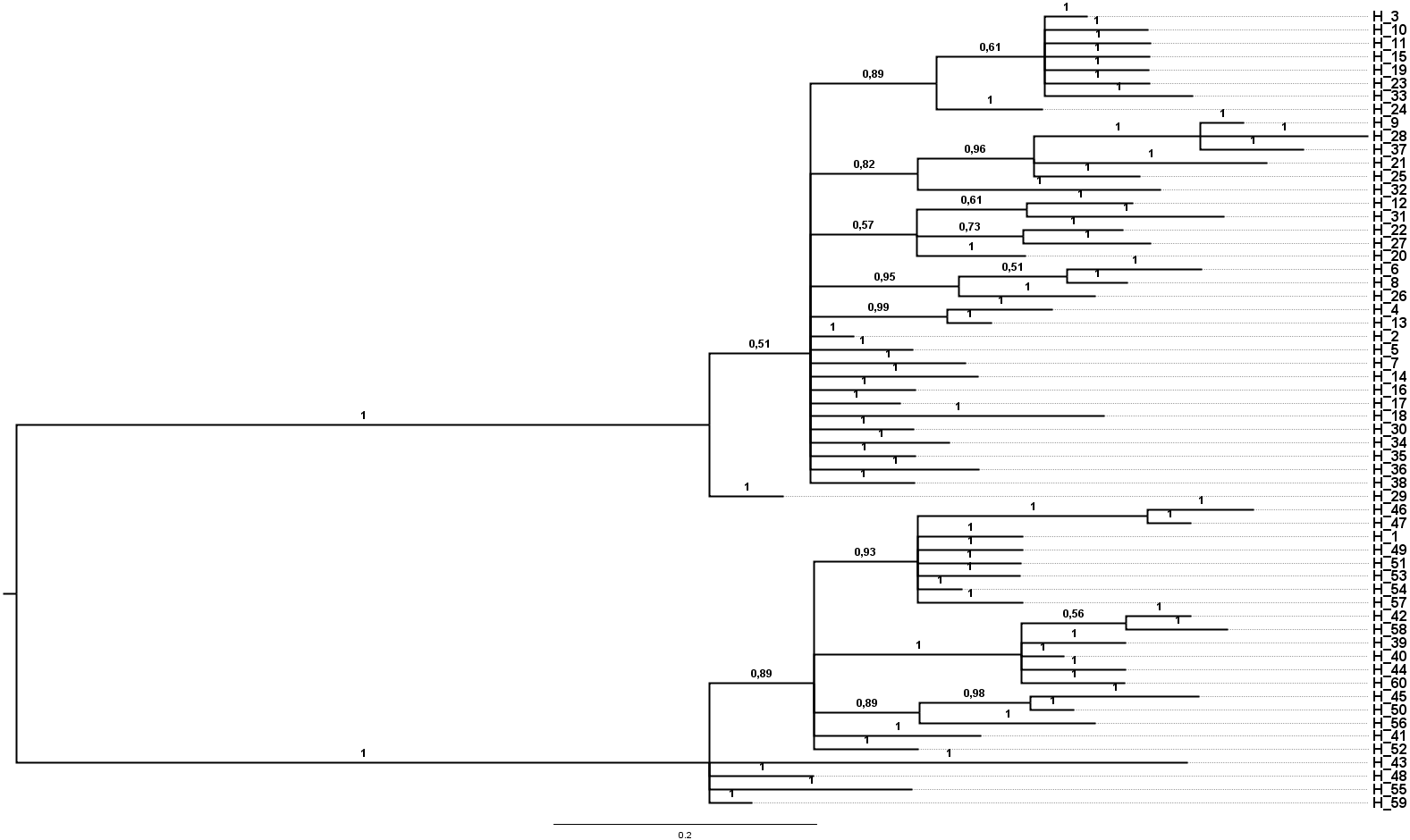


**Fig. S2-a**, *Tlr4* haplogroup definition; Haplotype network reconstructed in Network v. 4.6.1.1., phylogeny taken from Fig. S 1-a, distribution of haplotypes among sampled individuals can be found in Table S1.


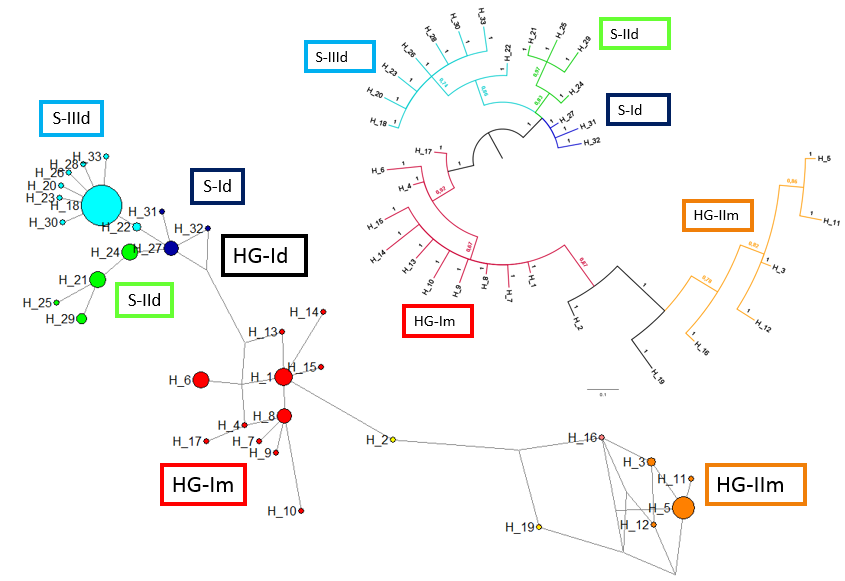


**Fig. S2-b**, *mt-Cytb* haplogroup definition; haplotype network reconstructed in Network v. 4.6.1.1., phylogeny taken from Fig. S 1-b, distribution of haplotypes among sampled individuals can be found in Table S1.


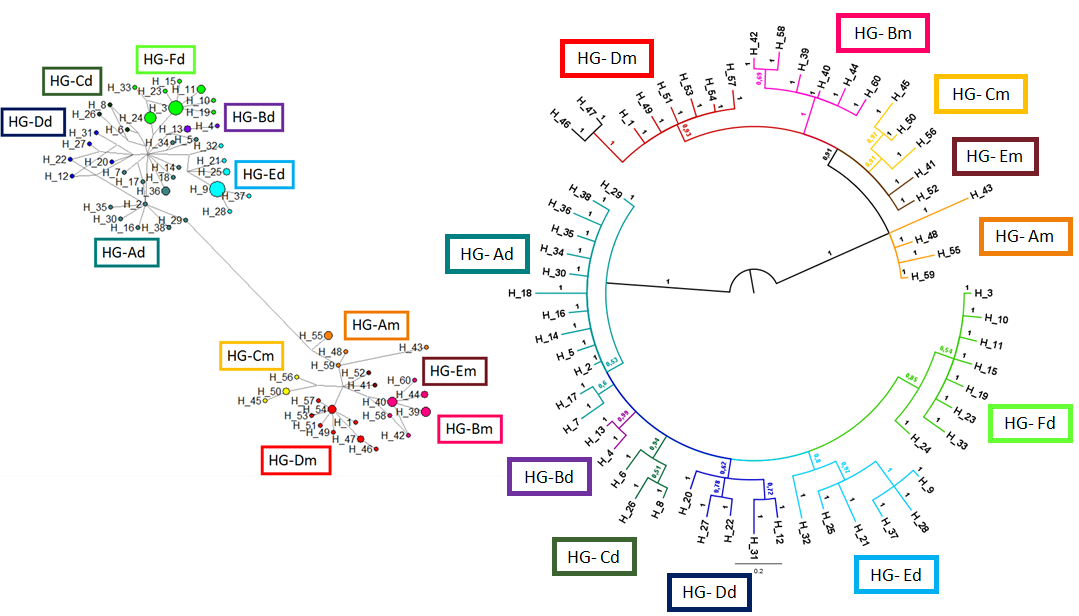


**Fig. S3**, Evidence of recombination between HG-Im and HG-IIm of Mmm, breakpoints in Mmm were detected in our alignment (a) by SBP at position 1326bp (529aa) and (b) by GARD at position 1350 (537aa), these breakpoints are represented by a vertical black dashed line between positions 1326 and 1350. RH, presumably recombinant haplotypes (H_2 and H_19) are separated by horizontal black dashed line from the rest of haplotypes. Varible sites were extracted by Fabox DNA collapser (Villesen 2007).


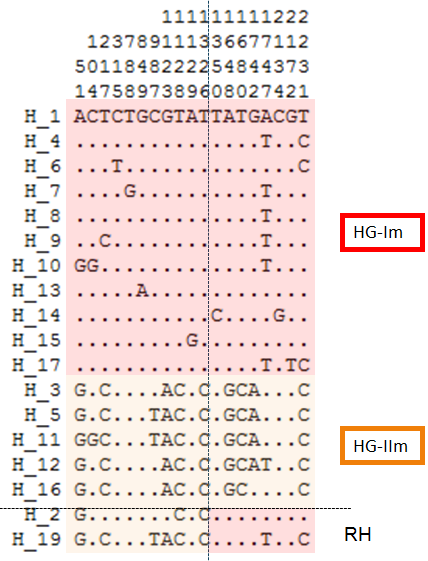

Supplement: Supplementary file 1 [file ece30004-2931-SD1.doc]
